# Functional test of a naturally occurred tumor modifier gene provides insights to melanoma development

**DOI:** 10.1093/g3journal/jkae298

**Published:** 2025-01-17

**Authors:** Mateo Garcia-Olazabal, Mateus Contar Adolfi, Brigitta Wilde, Anita Hufnagel, Rupesh Paudel, Yuan Lu, Svenja Meierjohann, Gil G Rosenthal, Manfred Schartl

**Affiliations:** Xiphophorus Genetic Stock Center, Texas State University, San Marcos, TX 78666, USA; Department of Cell and Developmental Biology, Biocenter, University of Würzburg, 97074 Würzburg, Germany; Department of Biochemistry and Cell Biology, University of Würzburg, 97074 Würzburg, Germany; Institute of Pathology, University of Würzburg, 97080 Würzburg, Germany; Department for Dermatology and Allergology, University Hospital Würzburg, 97080 Würzburg, Germany; Xiphophorus Genetic Stock Center, Texas State University, San Marcos, TX 78666, USA; Institute of Pathology, University of Würzburg, 97080 Würzburg, Germany; Centro de Investigaciones Científicas de las Huastecas, A.C. 43230 Calnali, Hidalgo, Mexico; Department of Biology, Università degli Studi di Padova, 35121 Padova, Italy; Xiphophorus Genetic Stock Center, Texas State University, San Marcos, TX 78666, USA; Department of Cell and Developmental Biology, Biocenter, University of Würzburg, 97074 Würzburg, Germany; Research Department for Limnology, University of Innsbruck, A-5310 Mondsee, Austria

**Keywords:** melanoma, tumor suppressor, Xiphophorus, hybrid

## Abstract

Occurrence of degenerative interactions is thought to serve as a mechanism underlying hybrid unfitness in most animal systems. However, the molecular mechanisms underpinning the genetic interaction and how they contribute to overall hybrid incompatibilities are limited to only a handful of examples. A vertebrate model organism, *Xiphophorus*, is used to study hybrid dysfunction, and it has been shown from this model that diseases, such as melanoma, can occur in certain interspecies hybrids. Melanoma development is due to hybrid inheritance of an oncogene, *xmrk*, and loss of a co-evolved tumor modifier. It was recently found that *adgre5*, a G protein-coupled receptor involved in cell adhesion, is a tumor regulator gene in naturally hybridizing *Xiphophorus* species *Xiphophorus birchmanni* (*X. birchmanni*) and *Xiphophorus malinche* (*X. malinche*). We hypothesized that 1 of the 2 parental alleles of *adgre5* is involved in regulation of cell growth, migration, and melanomagenesis. Accordingly, we assessed the function of *adgre5* alleles from each parental species of the melanoma-bearing hybrids using in vitro cell growth and migration assays. In addition, we expressed each *adgre5* allele with the *xmrk* oncogene in transgenic medaka. We found that cells transfected with the *X. birchmanni adgre5* exhibited decreased growth and migration compared to those with the *X. malinche* allele. Moreover, *X. birchmanni* allele of *adgre5* completely inhibited melanoma development in *xmrk*-transgenic medaka, while *X. malinche adgre5* expression did not exhibit melanoma suppressive activity in medaka. These findings provide evidence that *adgre5* is a natural melanoma suppressor and provide new insight in melanoma etiology.

## Introduction

Almost a century ago, it was discovered that certain hybrids of the southern platyfish *Xiphophorus maculatus* (*X. maculatus*) and green swordtails *Xiphophorus hellerii* (*X. hellerii*) develop highly malignant melanoma (Koßwig 1928; Haussler 1928) due to the inheritance of an oncogene, and conditional loss of a hypothetical tumor modulator (for review, see Schartl and Walter (2016) and Schartl and Lu (2024)). From this hybrid crossing experiment, a possible genetic explanation to how hybrid melanoma is generated is that certain individuals of the platyfish carry an oncogene modulated by a tumor suppressor. In this case, the oncogene effect on the platyfish is only a local dysplasia of melanocytes visible as a black pigment spot of macromelanophores. The oncogene and the tumor suppressor are located in different linkage groups, thus, when a platyfish is crossed with a swordtail that lacks both genes, some individuals will express the oncogene without the control of the tumor suppressor and therefore develop malignant melanoma.

It has been found that a mutant duplicate of *Xiphophorus* Epidermal Growth Factor Receptor (*egfrb*) named *Xiphophorus melanoma receptor kinase* (*xmrk*) is a bona fide oncogene driving the melanomagenesis observed in *Xiphophorus* hybrids, whose expression is necessary and sufficient for tumor development in *Xiphophorus* (Schartl *et al*. 1999, 2010; Schartl and Lu, 2024). The *xmrk*-driven melanoma is thought to be a postzygotic mechanism for species isolation, and the *xmrk* gene is an example of the few speciation genes identified to date (Schartl 2008; Flachs *et al*. 2012). However, other than tumor modifiers identified from laboratory interspecies hybrids, there is no real-life example showing that *xmrk* is involved in species isolation until a study recently identified natural hybrid zones involving *X. birchmanni* and X*. malinche* (Kazianis *et al*. 1998; Lu *et al*. 2020; Powell *et al*. 2020). *X. birchmanni* has *xmrk* and is polymorphic for a black spot in their caudal fin, comprised of hyperplastic melanophores. *X. malinche* lacks *xmrk* as well as the caudal fin black spot. Interspecies hybrids between the 2 can develop melanoma. Powell *et al*. (2020) performed an admixture mapping study of these hybrids and revealed that hybrid malignant melanoma occurs when the hybrid individuals inherited *xmrk* from *X. birchmanni* and the *X. malinche* allele of *adgre5* (Powell *et al*. 2020). The *adgre5* gene encodes a G protein-coupled receptor, with varied roles in cell adhesion and signaling (Yona *et al*. 2008). The human ortholog plays a vital role in the adhesion and migration of healthy immune cells, however, it is also a mediator of invasion in a variety of human cancers (reviewed in Safaee *et al*. (2013)). The *X. birchmanni* and *X. malinche adgre5* alleles are different by 5 amino acids, including 1 in a conserved epidermal growth factor-like calcium binding site (Powell *et al*. 2020), These evidences suggested that *X. birchmanni adgre5* is a *xmrk* regulator while the *X. malinche* allele is insufficient for this function*. adgre5* codes for protein CD97. It will be referred to as *adgre5* because it is the HUGO Gene Nomenclature Committee approved name.

Despite the finding that *adgre5* is associated to melanoma tumor suppression, there is no functional test to validate the activity of the *adgre5*. Therefore, this study aimed to test both in vitro and in vivo if the *X. birchmanni adgre5* can inhibit melanoma development. We hypothesize that *adgre5* affects melanocyte growth and migration. To test if *adgre5* can act as a general tumor suppressor of pigment cells, we expressed the *X. birchmanni* and *X. malinche adgre5* in mammalian melanocyte and assessed melanocyte growth and invasion. In addition, to test if *adgre5* can specifically suppress *xmrk*, we introduced both *adgre5* alleles separately into *xmrk-*transgenic medaka that develop pigment cell lineage-specific tumors to evaluate if the 2 parental species alleles are different in arresting *xmrk* oncogenicity.

## Methods

### Cell culture and generations of stable adgre5 expression lines

Murine melanocyte Melan-A cells were cultured in DMEM with pyruvate (Gibco #11995073), supplemented with 10% fetal calf serum, 1% penicillin/streptomycin, and maintained at 37°C, 5% CO_2_ with 100% humidity.

To generate doxycycline-inducible expression cell lines, the vector pSB-ET-iE (M. Gessler, Dept. of Developmental Biochemistry, University of Wurzburg) was used. It allows integration of genes by *sleeping beauty*-mediated transposition. Dox-inducible promoters enable precise regulation of the expression of the targeted gene, facilitating elegant experimental designs. Here, the responsive T6 promoter drives expression of *adgre5* and EGFP, with an IRES site between them (Supplementary Fig. 1a). After transfection, these cells were selected with 1 μg/ml puromycin for 2 weeks.

*X. malinche* and *X. birchmanni* alleles of *adgre5* were amplified using primers with XbaI and ClaI restriction enzyme sites (adgre5 for: CGTCTAGAATGCCCTTCTCTAATCTACACC, adgre5 rev: GCGCATCGATTCACTTATCGTCGTCATCCTTGTAATC). PCR amplification was performed using cDNA from tissue samples of organisms of *X. birchmanni* and *X. malinche*. The High Fidelity Q5 polymerase (New England Bio Labs #M0491S) was used for PCR amplification. The respective restriction enzymes were used to clone the PCR product into the vector, and wild-type Melan-A cells were transfected using Fugene transfection reagent (Promega Catalog #E5911). As a result, pSB-ET-iE_*X. malinche*_*adgre5* and pSB-ET-iE_*X. birchmanni*_*adgre5* stable cell lines were generated. Unfortunately, there is no antibody available for *Xiphophorus adgre5*. Previous experience in the usage of cross-species reactive antibodies has shown us that cross-species antibodies are only useful when the target protein antigen is a highly conserved primary sequence. We used GFP as an indicator of overexpression (Supplementary Fig. 2). No significant differences were observed in the expression levels of *adgre5* between Melan-A cell lines containing the different alleles (Supplementary Fig. 2, ANOVA *P* > 0.05 for differences between *X. birchmanni* and *X. malinche* adgre5 alleles).

### RNA extraction, cDNA synthesis, and qPCR

RNA extraction, cDNA synthesis, and qPCR were performed to measure the gene expression levels of *adgre5* in the Melan-A cell lines transfected with different *adgre5* alleles. Total RNA was extracted from freshly harvested cells or cell pellets that were stored at −80°C, using TRIzol reagent according to the manufacturer's protocol. DNase I digestion was carried out for 1 hour at 37°C to eliminate any contaminating DNA. RNA concentration was determined using a NanoDrop spectrophotometer. RevertAid First Strand cDNA Synthesis Kit and random hexamer primers were used to reversely transcribe 100–4,000 ng of RNA according to the manufacturer's instructions (Thermo Fisher Catalog #K1622). Fluorescence-based RT-qPCR was performed using SYBR Green reagent and analyzed with a Mastercycler ep Realplex. Gene expression levels were normalized to a housekeeping gene (*Hprt* fwd: ACTGGCAACACTAACAGGACT, *Hprt* rev: TGTTGTTGGATATGCCCTTG, *adgre5* fwd: CATCCGGCCCCTTTACTTGT, *adgre5* rev: GGGCCAAAGAAGCTCCAGAT) using the delta-delta Ct method, and the success of transfection and overexpression was confirmed by checking for GFP expression using an inverted fluorescence microscope with a 63× objective (Supplementary Fig. 1c). Three independent replicates of cells were used for each treatment and each cell line.

### Cell growth assay

In triplicate, cells were counted and seeded at equal density (1–2 × 10^3^ cells/well) in 96-well plates. Cells were treated with either dox 0 or dox 500 (500 mg/ml) by adding the respective dox concentrations to cell media. On days 2, 3, and 4 after treatment, 5 mg/ml of 3-(4,5-dimethylthiazol-2-yl)-2,5-diphenyltetrazolium bromide (MTT) was added to each well at a ratio of 1:5 (MTT:medium). The medium was aspirated after 2 hours of incubation at 37°C, and 150 µl of DMSO was added to each well. The plate was then incubated on a shaking device at room temperature for 15 minutes. A microplate reader was used to measure formazan accumulation by reading the optical density at 590 nm with a reference filter of 620 nm. Quintuples for each treatment, species, and duration of the experiments were performed. Cell growth was calculated by subtracting the optical density at 590 nm observed in dox 0 cells from the optical density at 590 nm observed in dox 500 induced cells. An ANOVA and a Tukey post hoc test were used to determine the statistical differences between *adgre5 X. birchmanni* and *X. malinche* alleles in cell growth.

### Trans-well migration assay

To assess cell migration, cells previously starved in 1% dialyzed fetal calf serum (FCS) for 24 hours were seeded at 2 × 10^3^ per well in the upper layer of uncoated trans-well inlays with 8 µm pore diameter in 24-well plates. The cells were treated with either dox 0 or dox 500 (500 mg/ml) for 24 hours. To stimulate migration, medium containing 10% FCS and either dox 0 or dox 500 (500 mg/ml) was added to the lower layer of the trans-well, and the cells were allowed to migrate for 16 hours. Each assay was performed in triplicate per cell line and per dox treatment. Non-migrated cells were removed by cotton swabs, and the migrated cells were fixed with methanol, stained with 0.2% crystal violet dye in 2% ethanol for 15 minutes, and washed with PBS. The membrane was cut out of the trans-well inlay, embedded with Mowiol (polyvinyl alcohol) on microscope slides, and images were captured. The migrated cells were counted under a microscope, and migration was calculated as the difference in the number of cells that migrated in dox 500-induced cells minus the number of cells that migrated in dox 0 cells.

### Isolation of the *Xiphophorus adgre5* and construction of expression vectors with *fugu tyrp* promoter

The *adgre5* was designed to be driven by a pigment cell specific promoter, *Fugu rubripes* Tyrosinase-related Protein 1 gene (*tyrp*) (del Marmol and Beermann 1996; Zou *et al*. 2006). To isolate the *adgre5* gene from *Xiphophorus*, a high-fidelity PCR (using Q5 Taq enzyme) was performed from cDNA extracted fin tissue from either *X. birchmanni* or *X. malinche* using overhang primers containing the XbaI restriction enzyme cutting site (for: GCGTCTAGAATGCCCTTCTCTAATCTACACC, rev: GCGCTCTAGATCATATTTGGGATTCTCCTGTGATCT). Each cloning step was performed according to manufacturer's protocols and plasmids were extracted using Qiagen Miniprep Kit. Plasmids were sequenced to verify that no mutations occurred during cloning.

mCerulean was cut out with its promoter and a bGH poly(A) site using KpnI restriction enzyme from the brainbow vector (Livet *et al*. 2007). The pIsceI-typr-pa 3 plasmid containing the *tyrp fugu* promoter was linearized with KpnI restriction enzyme and ligated to mCerulean. The resulting vector was subsequently digested with XbaI and ligated to the previously XbaI-digested *adgre5* PCR products. Thus, specific plasmids were created containing either the *X. malinche* or *X. birchmanni* alleles of *adgre5* under the expression of the *tyrp fugu* promoter (Supplementary Fig. 3)

### Generation of transgenic medaka

To generate stable transgenic lines, the meganuclease injection protocol was used since it was demonstrated to be more effective than injecting the plasmid alone, as it reduces mosaic expression, increases frequency of positive founder fish, and increases germline transmission rates (Thermes *et al.* 2002). One-cell stage *tg*(*mitf*:*xmrk*) medaka embryos (*Oryzias latipes* strain: Carbio, homozygous for *xmrk*) were injected into the cytoplasm with approximately 15–20 pg of total DNA plasmid in a volume of 500 pl injection solution containing I-SceI meganuclease. Adult F0 fish were mated with each other and the offspring were tested for the presence of the transgene by screening of blue eyes under UV light (mCerulean effect).

All animal studies have been approved by the authors’ Institutional Review Board (Animal Welfare Officer of the University of Wurzburg). Adult fish were maintained under standard conditions with an artificial photoperiod (10 hours of darkness, 14 hours of light) to induce reproductive activity. Clusters of fertilized eggs were collected 0.5–1 hour after the onset of light and kept in a rearing medium containing 0.1% NaCl, 0.003% KCl, 0.004% CaCl_2_ × 2H_2_O, 0.016% MgSO_4_ × 7H_2_O, and 0.0001% methylene blue.

F1 fish (8 *tg*(*mitf*:*xmrk) + tyrp:Xmal* and 7 *tg*(*mitf*:*xmrk) + tyrp:Xbir*) were anesthetized in MS-222 and photographed with a Nikon D300 digital camera with a Tamron SP 90 mm F/2.8 1:1 Macro lenses. Individuals were quantified for hyperpigmented melanic areas from the images using ImageJ (Abramoff et al. 2004; Rasband 1997–2018; Schneider *et al.* 2012). Data fitted normality principles and therefore were analyzed with an ANOVA and a Tukey post hoc test.

## Results

### *X. malinche* but not *X. birchmanni* allele of *adgre5* promotes migration

**Fig. 1.**
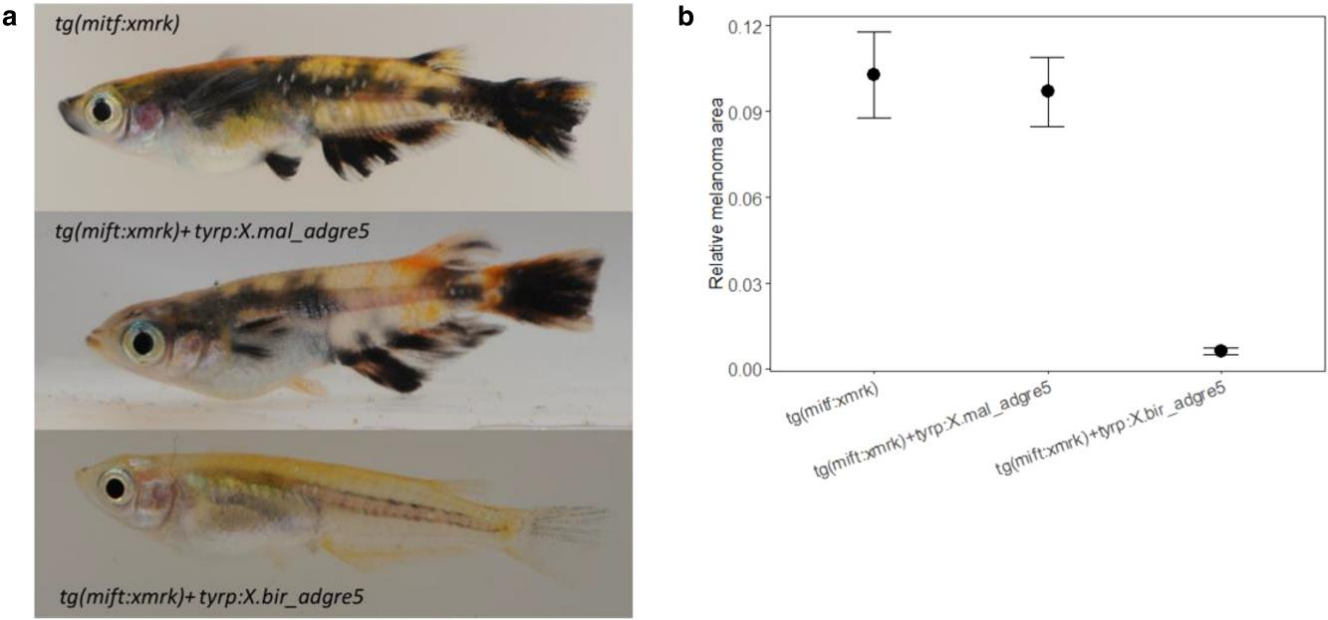
a) Migration assay of cells expressing different *adgre5* alleles. *Y*-axis indicates the relative migration calculated as the difference between number of cells that migrated in dox 500 induced cells minus the number of cells that migrated in dox 0 cells. Xmal = stable cell line transfected with the *X. malinche* allele of *adgre5*. Xbir = stable cell line transfected with the *X. birchmanni* allele of *adgre5*. pSB-ET-iE is the control vector. Each assay was performed in triplicate per cell line. b) Growth of cells expressing different *adgre5* alleles. *Y*-axis indicates relative growth calculated as the difference in optical density at 590 nm observed in dox 500 induced cells minus the optical density at 590 nm observed in dox 0 cells in the MTT assay for each cell line and for each duration of the growth experiment. Quintuples for each treatment, species, and duration of the experiments were performed. The plots show the mean, and whiskers indicate 2 SEM.

To assess the potential role of *adgre5* in promoting cell migration, we performed trans-well migration assay of Melan-A cells expressing *X. birchmanni* and *X. malinche adgre5*, respectively. Melan-A cells expressing the *X. birchmanni* allele of *adgre5* did not show difference in migration propensity compared to the control vector transfected cells. However, cells expressing the *X. malinche* allele of *adgre5* exhibited enhanced migration compared to both control cells and cells expressing *X. birchmanni adgre5* (Fig. 1a, *t*-test *P*-value = 0.00582. Control (pSB-ET-iE): mean= 27.7, relative migration SD = 4.04, Xbir_adgre5: mean = 24.6, relative migration SD = 6.11, Xmal_adre5: mean = 124.6, relative migration SD = 17.9. Tukey post hoc test < 0.05).

### *X. birchmanni* allele of *adgre5* inhibits pigment cell growth

Melan-A cells transfected with the *X. birchmanni* allele of *adgre5* showed lower cell numbers than control cells transfected with empty vector after 3 and 4 days of expression induction. In comparison, the cells expressing *X. malinche* adgre5 also exhibited lower cell number than control cells, but its effect is less potent than the *X. birchmanni* allele (Fig. 1b, ANOVA *P* < 0.0001, Tukey post hoc *P*-values, day 2: *X. birchmanni* vs *X. malinche P* = 0.49; day 3: *X. birchmanni* vs *X. malinche P* = 0.0006; day 4: *X. birchmanni–X. malinche* day 4 *P* = 0.0019. Mean and SD available in Supplementary Table 1).

### *X. birchmanni adgre5* eliminates *xmrk-*driven melanomagenesis in transgenic medaka

**Fig. 2.**
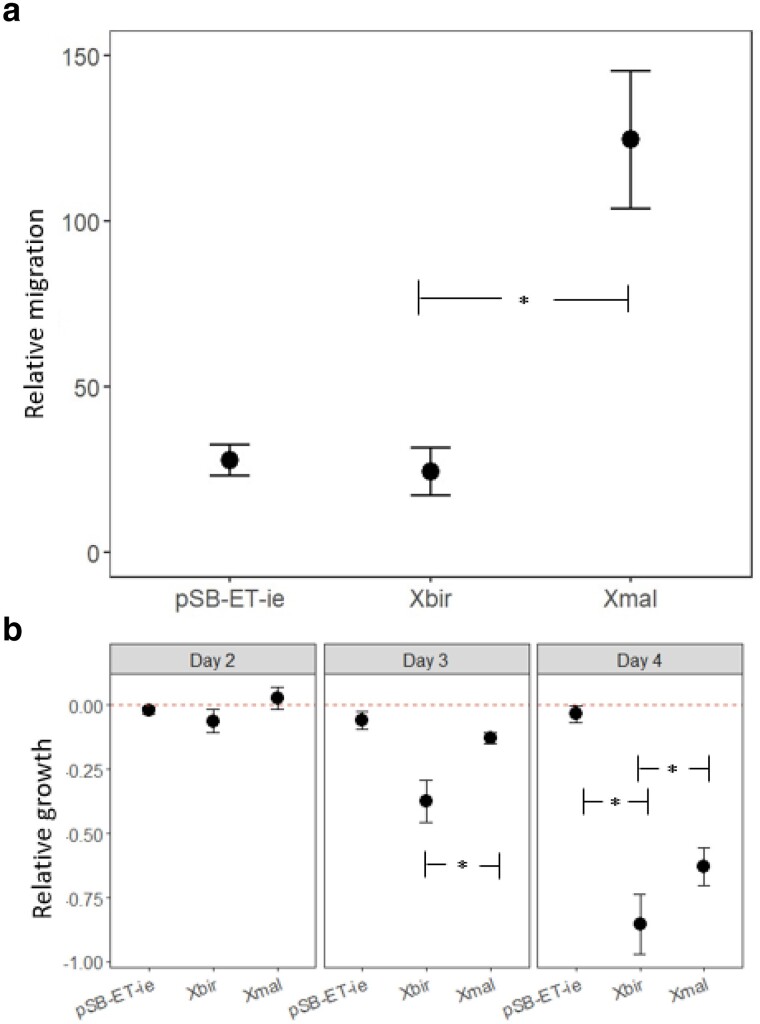
a) Phenotypes of the single and double transgenic fish. All individuals photographed of *tg(mift:xmrk) + tyrp:X.mal_adgre5*, *tg(mift:xmrk) + tyrp:X.bir_adgre5* are F0s, *tg(mitf:xmrk)* are more than 30 generations old. b) Relative melanoma covered area (corrected by standard length) for each transgenic line generated (*tg(mift:xmrk)* N = 20, *tg(mift:xmrk) + tyrp:X.mal_adgre5* N = 5, *tg(mift:xmrk) + tyrp:X.bir_adgre5* N = 5). The plot shows the mean, and whiskers indicate 2 SEM.

*Xiphophorus* is viviparous. Despite efforts are being put into development of producing transgenic *Xiphophorus* lines, there is currently no established protocol for this task. Therefore, we utilized an egg-laying fish model organism that is closely related to the *Xiphophorus.* A transgenic medaka line has been developed with a homozygous knock-in of *Xiphophorus xmrk* expressed under control of the medaka *mitf* promoter *(ref)*. This line develops pigment cell lineage-specific tumors with 100% genetic penetrance, and has been shown to be a valid model system to test gene functions for arresting the *xmrk* oncogenic activity (Elizabeth Patton and Nairn 2010; Schartl *et al*. 2010; Klotz *et al*. 2018; Sugiyama *et al*. 2019; Regneri *et al*. 2019; Abdulsahib *et al*. 2024). We expressed *X. birchmanni* and *X. malinche* alleles of *adgre5* in *xmrk-*transgenic medaka generating the double transgenic medakas, respectively, *tg(mift:xmrk) + tyrp:X.bir_adgre5*, and *tg(mift:xmrk) + tyrp:X.mal_adgre5* (the *tg(mift:xmrk)*) indicates that the construct was injected into an already genetically modified medaka line carrying *xmrk*. The *+tyrp:X.bir_adgre5* indicated the construct we injected to generate the double transgenic). The *tg(mift:xmrk) + tyrp:X.bir_adgre5* showed significant reductions of pigmentation compared to control *tg(mift:xmrk)* fish (Fig. 2; *P* < 0.00001), or *tg(mift:xmrk) + tyrp:X.mal_adgre5* medaka (Fig. 2; *P* < 0.00001), and devoid of pigment cell tumor. In comparison, the *tg(mift:xmrk) + tyrp:X.mal_adgre5* and *tg(mitf:xmrk)* medaka lines did not show significant differences in pigmentation to *xmrk* control medaka (Tukey post hoc *P*-values, *tg(mift:xmrk) + tyrp:X.mal_adgre5*-*tg(mitf:xmrk)* = 0.85). While there is an effect of *X. birchmanni adgre5* on the *xmrk* transformed macromelanophores, we noticed no effect on the medaka micromelanophores which are responsible for the normal body pigmentation.

## Discussion

This study focuses on functionally testing the role of the *X. birchmanni* and *X. malinche adgre5* alleles by analyzing their effect on cell growth and migration and melanoma development. Melan-A cells are a well-established line of normal mouse melanocytes (Rossato *et al*. 2014) and provide an excellent non-tumorigenic line for studying the cellular and molecular basis of melanoma malignancy (Bennett *et al*. 1987), particularly for studies aimed at understanding the mechanisms triggering the change from benign pigmentation lesions to malignant melanoma. Two main characteristics of cancer are unlimited cell growth and potent metastatic properties (Warburg 1956). Therefore, it is expected that tumor suppressor genes cause a decrease of cell growth and lowered propensity in migration. Even though melanoma modifying genes are easiest to investigate in cell culture models, animal models are imperative to evaluate their importance in the context of the whole organism. Transgenic organisms isolate effects of the induced gene from other unknown possible genetic interactions. The transgenic medaka melanoma perfectly mirror the situation described for the Xiphophorus tumors on the cell biological and molecular level (for cell biology, see Regneri *et al*. (2019) and for human comparative transcriptomic, see Schartl *et al*. (2012) and Mishra *et al*. (2014)). The lack of differences in the areas covered by melanoma cells between the *tg(mift:xmrk) + tyrp:X.mal_adgre5* and *tg(mitf:xmrk)*, as well as the striking reduction of pigmentation of *tg(mift:xmrk) + tyrp:X.bir_adgre5*, suggests that *adgre5* acts indeed as a tumor suppressor. The double transgenic medaka expressing *xmrk* and *adgre5* in pigments cells shows only benign, premalignant pigment lesions (see Fig. 2a bottom) that are comparable to the nevi of mammals (for a characterization of these premalignant lesions, see Regneri *et al*. (2019)). Thus, we hypothesize that the tumor suppression phenotype occurs at very early stages of tumorigenesis rather than a later inhibition of migration. Due to transgene integration site, methylation effects and other parameters known to modify expression of transgenes a larger number of *adgre5 xmrk* double transgenics would be useful in the future.

Consistent with these in vivo results on melanoma growth, pigment cells transfected with the *X. birchmanni* allele of *adgre5* caused cells in culture to grow slower. Moreover, the *X. birchmanni* allele of *adgre5* did neither enhance nor inhibit cell migration, however, the *X. malinche* allele enhanced migration; a typical quality of invasive cancerous cells. These results are also consistent with the results found in natural hybrids of *X. birchmanni* and *X. malinche* where homozygous *X. malinche adgre5* showed the most malignant phenotypes (Powell *et al*. 2021)

*ADGRE5* is a prototypic member of the adhesion class of G protein-coupled receptors (adhesion GPCRs), which plays vital roles in numerous developmental processes as well as in tumorigenesis. Although it has been demonstrated that *adgre5*, under apoptotic conditions, can increase tumor cell viability by inhibition of caspase activation and modulation of anti- and pro-apoptotic members of the BCL-2 superfamily (Hsiao *et al*. 2015), it has also been demonstrated that G protein-coupled receptors inhibit melanoma tumor growth and metastasis (Xu *et al*. 2006). In fact, a recent review (Langenhan 2020) proposes adhesion G protein-coupled receptors as candidate metabotropic mechanosensors and novel drug targets for numerous cancer types because they are surface molecules that act as mechanosensors. Information about *adgre5* function could also be expected from loss of function studies. Recently, it was demonstrated that *xmrk* effect can be suppressed when co-expressed with a known potent tumor suppressor in medaka double transgenic lines: *cdkn2ab* (Regneri *et al*. 2019). Future studies to further characterize the role of *adgre5* as a tumor suppressor are needed. For example, a tissue-specific knockout of medaka *adgre5* could cause even more malignant melanoma phenotypes, in analogy to the knockout of *cdkn2ab* which resulted in strongly enhanced tumor growth (Regneri *et al*. 2019).

The study by Powell *et al*. (2020) was the first to propose a candidate hybrid incompatibility identified to the single-gene level in naturally occurring hybrids. Almost simultaneously, another important example of hybrid incompatibility single-gene-level identification came from the closely related artificial hybrids between *X. maculatus* and *X. hellerii* (Powell *et al*. 2020). Lu *et al*. (2020) successfully identified *rab3d* as a tumor suppressor candidate gene. Future studies should focus on experimentally testing the role of *rabd3* (Lu *et al*. 2020) in a similar approach as taken here for *adgre5* using transgenic studies with the medaka tg(mitf:xmrk) line as well as cell culture experiments.

The results of both studies are interesting because they propose that alleles of different genes, 7 Mb apart on the same chromosome, which interact with *xmrk*, are responsible for hybrid incompatibility in 2 different pairs of species from the same genus *Xiphophorus*. This suggests that a melanoma incompatibility involving *xmrk* originated independently in 2 distinct lineages. Schartl (2008) proposed that *R* (as it was called the then unknown tumor regulator gene) “could have preexisted before *xmrk* arose and would have suppressed the melanoma from the moment when the oncogene arose”. The most parsimonious hypothesis is that *xmrk* originated in a common ancestor to all *Xiphophorus* and that it has been repeatedly lost in several branches of the tree. In contrast, it appears that all species have *R*, but different alleles, because different hybrid crosses have different levels of *xmrk* suppression (Atz 1962; Anders 1967). Indeed, the recent findings suggest that R “different alleles” are actually different genes.

There has been renewed interest in the search for Bateson–Dobzhansky–Muller hybrid incompatibilities since we realized that hybridization is more common than previously thought (Jhonson 2002; Schumer *et al*. 2014). However, few studies have been able to precisely identify the interacting genes responsible for the incompatibility (Presgraves 2010). Of those that have, most identify candidate genes by using hybrids between crosses of model species that no longer hybridize naturally (Lee *et al*. 2008; Bikard *et al*. 2009; Yu *et al*. 2018; Zuellig and Sweigart 2018). The list of studies that have been able to pinpoint single gene effects grows significantly when considering which ones are actually able to experimentally test them and effectively assign a causal relationship between the gene interaction and the hybrid incompatibility (Mihola *et al*. 2009; Trachtulec *et al*. 2008).

To the best of our knowledge, the work presented here is the only study so far that has experimentally validated a candidate gene involved in a hybrid incompatibility in species that currently hybridize naturally. Further studies of this kind could shed important light on how incompatibilities affect speciation, specifically whether mapped incompatibilities trigger divergence between species or arise after gene flow has ceased. The results of this study provide strong evidence that *adgre5* acts as a tumor modifier and highlight the importance of following population genetic mapping studies with functional cell culture and in vivo studies. Future research focused on characterizing the specific mechanism by which a*dgre5* suppresses *xmrk* holds great promise for biomedical research. In 2024, about 100,640 new melanomas are expected to be diagnosed (about 59,170 in men and 41,470 in women), and about 8,290 people are expected to die of melanoma (about 5,430 men and 2,860 women) in the United States due to melanoma, which accounts for the vast majority of skin cancer deaths (American Cancer Society, 2024). Insights into how a tumor suppressor gene can counteract an oncogene will be of tremendous value in the development of melanoma treatments.

## Supplementary Material

jkae298_Supplementary_Data

## Data Availability

The authors affirm that all data necessary for confirming the conclusions of this article are represented fully within the article and its tables and figures. Supplemental material available at G3 online.
