# Supplementary material for: Functional test of a naturally occurred tumor modifier gene provides insights to melanoma development
Source: G3 (Bethesda). 2025 Jan 17;15(2):jkae298. doi: 10.1093/g3journal/jkae298 (PMC11797068; doi:10.1093/g3journal/jkae298)
Supplement: jkae298_Supplementary_Data [file jkae298_supplementary_data.docx]

Supplemental figures & Tables


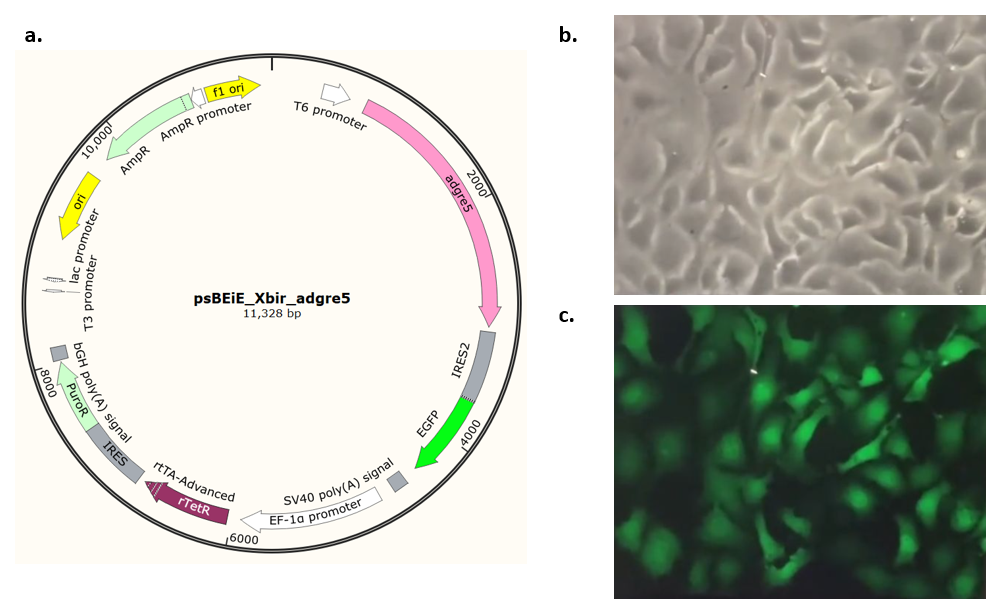


**Figure S1.** a) Map of the used for cell transfections. *X. birchmanni* and *X. malinche* plasmids are exactly the same except ORF of the *adgre5* which corresponds to each species. b) Bright field view of melan-a cells under an inverted microscope with a 63x objective c) Fluorescence image of b).


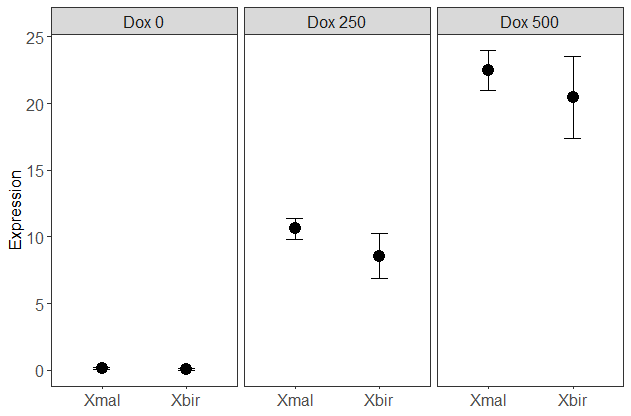


**Figure S2.** qPCR data of *adgre5* expression relative to the housekeeping gene *hprt* in melan-a cells after one day of dox induction. Xmal = stable cell line transfected with the *X. malinche* allele of *adgre5*. Xbir = stable cell line transfected with the *X. birchmanni* allele of *adgre5*. The number besides Dox indicates how many mg/ml of Dox were added to cell culture media. The plot shows the mean, and whiskers indicate two standard errors of the mean.


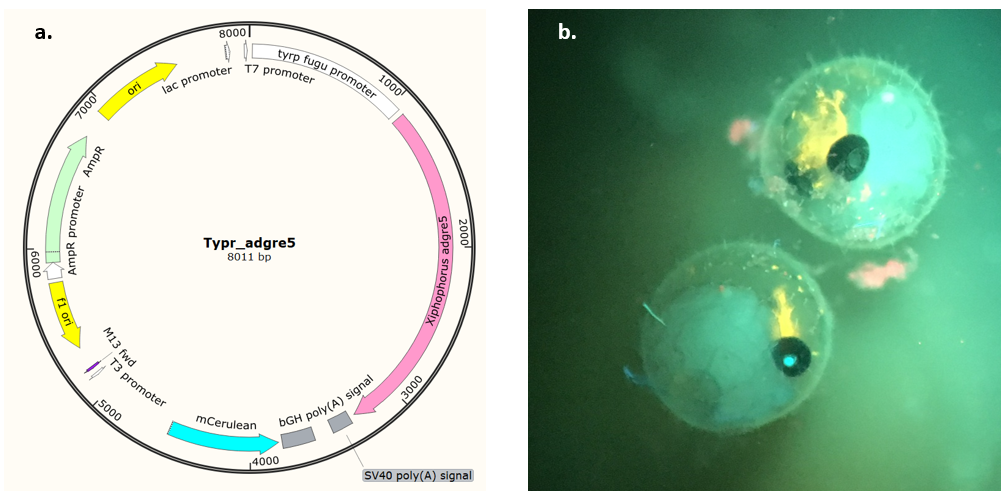


**Figure S3.** a) Map of the used for embryo injections. *X. birchmanni* and *X. malinche* plasmids are exactly the same except ORF of the *adgre5* which corresponds to each species. b) mCelurean effect: when DNA is successfully integrated blue eyes (bottom) are visible. Wild type embryo (top).

**Table S1.** Mean and Standard Deviation for each cell type and each duration of the relative cell growth calculated as as the difference in optical density at 590 nm observed in dox 500 induced cells minus the optical density at 590 nm observed in dox 0 cells.

| Cell type | Days of treatment | Mean | St Dev |
| --- | --- | --- | --- |
| pSB-ET-ie | 2 | -0.0206 | 0.013 |
| pSB-ET-ie | 3 | -0.0586 | 0.0367 |
| pSB-ET-ie | 4 | -0.0348 | 0.0351 |
| Xbir_adgre5 | 2 | -0.0628 | 0.0505 |
| Xbir_adgre5 | 3 | -0.375 | 0.0915 |
| Xbir_adgre5 | 4 | -0.853 | 0.131 |
| Xmal_adgre5 | 2 | 0.0268 | 0.0496 |
| Xmal_adgre5 | 3 | -0.127 | 0.0254 |
| Xmal_adgre5 | 4 | -0.629 | 0.0815 |
